# A further move into electronic publishing

**DOI:** 10.1080/03009730802700564

**Published:** 2009-02-04

**Authors:** Arne Andersson, Gunnar Ronquist, Torbjörn Karlsson

Perhaps some of you remember an editorial we wrote for our journal almost ten years ago entitled ‘On the revival of an old and venerable journal’. We gave reasons for why our society should go on publishing Upsala Journal of Medical Sciences (UJMS) given the premises ‘that it is not free of charge to run a business of this kind, despite the fact that the journal has grown thinner over the last couple of years’. We argued that the most relevant move to make should be to have the journal published also by electronic means. We also gave measures on how to increase the number of good submitted manuscripts. The final statement of that editorial was: ‘we don't intend to perish’.

Well, what happened? The articles showed up in a fairly short time on the web site of UJMS, and anyone interested could read them due to the open access facility that we offered. The problem, however, was that the abstracts and full papers had to be easily recognized at for instance PubMed. We have struggled quite a lot with this extremely important aspect of electronic publishing, and with that experience in mind we recently decided to go for a publisher with broad expertise in the field. And that is why you are just now viewing an extensively updated issue of our journal. We dared to take this step, since we have experienced over the last years a significant increase of the number of submitted manuscripts (almost 100 papers the year 2008), the number of total citations according to Journal Citation Reports reaching an all-time high (229 citations last year), and the number of hits in PubMed for the month of June last year exceeding 1500 (usually around 500 a couple of years ago). Due to the increased submission rate our acceptance rate for the submitted manuscripts has decreased well below 50%. And the impact factor has steadily increased at least over the last 4 years, but still it can be much improved.

We recommend that you all visit the new web site of our journal, www.informaworld.com/ujms. All manuscripts will now be handled online-only through Manuscript Central. This will further improve our manuscript handling and the quick electronic release (iFirst) of accepted papers. There will be a printed version as well, as you can easily realize from holding the latest issue in your hands. In fact we have been brave enough to increase the publication rate from three to four issues per year. Most importantly, we have decided to stay with the open access offer to all interested readers. Moreover, giving up delivering the articles in printed form has not been an option keeping in mind that each time our members receive their private copy it is a sign of the activity of their society. As can be seen from the slightly modified *Aims and Scope* of the journal we will continue our efforts to publish reports on both clinical and experimental work. We have, however, now stressed that contributions dealing with aspects of medical sciences that are frequently challenged by researchers at our own faculty are especially welcomed. That of course also means that we would like to encourage all our scientists, young and old, to submit some of their papers to our journal.

We didn't perish. Rather, we intend to serve as an efficient contributor of medical science reports.

